# Spontaneous refractive error, ocular biometry and age related lens changes in a population of geriatric rhesus macaques

**DOI:** 10.1038/s41598-025-30581-6

**Published:** 2025-12-05

**Authors:** Jonathon M. Ross, Karolina Roszak, Ana Ripolles-Garcia, Glenn Yiu, Christopher J. Murphy, Ala Moshiri, Hidetaka Miyagi, Ann R. Strøm, Soohyun Kim, Sangwan Park, M. Isabel Casanova, Lawrence S. Morse, Ariana Marangakis, Connor Chang, Laura M. Garzel, Jeffrey A. Roberts, Sara M. Thomasy

**Affiliations:** 1 Department of Surgical and Radiological Sciences, School of Veterinary Medicine, University of California Davis, 1 Shields Ave., Davis, CA 95616 USA; 2 Department of Ophthalmology & Vision Science, School of Medicine, University of California Davis, Sacramento, CA USA; 3 California National Primate Research Center, Davis, CA USA

**Keywords:** Refractive error, Ocular biometry, Nuclear sclerosis, Geriatric, Rhesus macaques, Cataract, Zoology, Anatomy, Ageing

## Abstract

The purpose of this investigation was to determine refractive error, ocular biometry and age-related lens changes in a population of geriatric rhesus macaques (*Macaca mulatta*) from the California National Primate Research Center (CNPRC). Ophthalmic examination was performed in 182 rhesus macaques $\:\ge\:$ 19 years of age using a cross-sectional study design, including streak retinoscopy, anterior segment tomography, A-scan ultrasound biometry and handheld slit lamp biomicroscopy. Median spherical equivalent refractive error was + 0.75 D with an interquartile range (IQR) of 0 to 1 D. Most eyes were hyperopic (*n* = 102, 55%) or emmetropic (*n* = 68, 36%); myopic eyes were the least common (*n* = 17, 9%). Anisometropia was present in 13 subjects (14%). Mean (± SD) corneal curvature was 52.6 ± 2.6 D (*n* = 79). Mean (± SD) axial globe length was 20.2 ± 1.5 mm, anterior chamber depth was 3.7 ± 0.4 mm, lens thickness was 4.1 ± 0.4 mm, and vitreous chamber depth was 12.2 ± 1.0 mm (*n* = 86). Median (IQR) nuclear sclerosis grade (*n* = 191, 98%) assessed with the lens opacities classification system II was 1 (1–2). Hyperopia is the most common refractive error in the geriatric rhesus macaque population at CNPRC. This study provides reference values for an isolated geriatric rhesus macaque population and broadens our understanding of refractive error and lens opacities in geriatric rhesus macaques which may serve as a model for studying novel therapeutics for refractive errors and cataracts.

**Supplementary Information:**

The online version contains supplementary material available at 10.1038/s41598-025-30581-6.

## Introduction

Non-human primates (NHPs) have proven to be useful models for studying the human visual system^1^. The genus *Macaca* is the most widely utilized NHP model and shares > 90% DNA sequence similarity to humans with comparable ocular size, structure and function^2^. Rhesus macaques (*Macaca mulatta*) have a lifespan of ~ 27 years in captivity while aging at a rate nearly 3 times that of humans^3,4^. In general, this species reaches adulthood (i.e. sexual maturity) by 5 years and is considered geriatric at ≥ 19 years of age^5^. Rhesus macaques demonstrate age-related ocular changes similar to humans, including development of cataracts^5,6^. Analysis of spontaneous changes in the geriatric rhesus macaque population may provide better understanding of the natural progression of aging in the human visual system.

Rhesus macaques raised in research facilities are often the subjects of translational investigations. Due to their similarities with humans, this species has proven to be invaluable for understanding refractive development and ocular changes observed in humans. However, to appreciate the full implication of the findings from studies in rhesus macaques a complete longitudinal observation (birth to geriatric) is necessary. To date, there have been few studies reported in the literature on refractive error in the geriatric rhesus macaque population. (Table 1). Thus, it is important to establish refractive error and ocular biometry characteristics across the lifespan of this species, particularly geriatric rhesus macaques.

**Table 1:** Summary of published studies on refractive error in rhesus macaques.

	Study	Location	Total rhesus macaques	Age (years)	Summary findings
1	Fernandes AG et al. 2023^64^	CPRC (free ranging)	120	Birth to 29	Spherical equivalent (mean ± SD): 0.30 ± 1.7 D (emmetropia ± low hyperopia/myopia)
2	Ma Y et al. 2023^65^	AAALAC (unknown)	219	8–21	Emmetropes and hyperopes: 38% Myopes: 62%
3	Zeng B et al. 2021^66^	GXBC or ZCBC (indoor)	15	12.4–16.1	Emmetropes and hyperopes: 98% Myopes: 2%
4	Qiao-Grider Y et al. 2007^11^	Indoor nursery	214	0.07–5	Spherical equivalent (mean ± SD): 4.17 ± 1.52 D (moderate and high hyperopia)
5	Fernandes A et al. 2003^34^	YNPRC (variable)	111	5–31	Spherical equivalent (mean ± SD): -0.19 ± 3.35 D (emmetropia ± moderate hyperopia/myopia)
6	Smith EL 3rd et al. 1999^67^	Indoor nursery	121	0.04–2	Spherical equivalent (mean): 4.4 D (moderate hyperopia)
7	Bradley DV et al. 1999^10^	YNPRC (unknown)	237	Birth to 5	Spherical equivalent (mean ± SD): 2 ± 1.2 D (low and moderate hyperopia)
8	Young FA. 1964^9^	WNPRC (unknown)	1000	0.7–20	Spherical equivalent (mean): 0.21 D (emmetropia)
9	Current study 2024	CNPRC (indoor & outdoor)	95	19–29	Emmetropes: 36% Hyperopes: 55% Myopes: 9%

CPRC, Caribbean Primate Research Center; AAALAC, Association for Assessment and Accreditation of Laboratory Animal Care; GXBC or ZCBC, Guangzhou Xiangguan Biotech Co., Ltd. or Zhaoqing Chuangyao Biotech Co., Ltd.; YNPRC, Yerkes National Primate Research Center; WNPRC, Washington National Primate Research Center; CNPRC, California National Primate Research Center. Data are presented as mean ± SD (refractive condition) unless otherwise indicated.

Mean refractive error distribution changes with age^7,8^. ​Previous investigations have determined refractive status and anterior segment biometrics in rhesus monkey populations from birth to adulthood^9–11^. Longitudinal studies in rhesus macaques from infancy to adulthood have reported measures of ocular biometry, including corneal curvature, endothelial cell density, axial length (AL), anterior and vitreous chamber depth (ACD and VCD, respectively) and lens thickness (LT)^12–19^. The incidence of cataract in rhesus macaques has also been investigated^6^. Uno and colleagues reported that 20% of postmortem rhesus macaque eyes (*n* = 175) had cataract at 20–22 years of age with significant increases after 26 years of age; although standardized grading was not performed^6^. By contrast, a lack of lens haze, cloudiness or age-related cataract was documented by Denlinger and coauthors in adult rhesus macaques,^20^ with only one primate being affected by a rosette shaped cataract of suspected traumatic etiology.

Despite detailed categorization of visual and ocular characteristics in young and adult rhesus macaques, there remains limited reports among the geriatric population in this species. The California National Primate Research Center (CNPRC) maintains a population of rhesus macaques ≥ 19 years of age so that aging studies can be conducted. Thus, a dataset comprised of geriatric refractive and ocular characteristics in rhesus macaques at CNPRC will provide reliable reference for future investigations involving both anterior and posterior segment anatomy and physiology and provide completeness to existing datasets on the natural progression of changes. The purpose of this investigation was to determine refractive error, ocular biometry and age-related lens changes in the eyes of geriatric (≥ 19 years of age) rhesus macaques using a cross-sectional study design.

## Methods

### Subjects

All rhesus macaques (*Macaca mulatta*) included in the investigation were maintained at the CNPRC during the study period. The CNPRC is accredited by the Association for Assessment and Accreditation of Laboratory Animal Care (AAALAC) International. Guidelines of the Association for Research in Vision and Ophthalmology Statement for the Use of Animals in Ophthalmic and Vision Research were followed. All aspects of this study were in accordance with the National Institutes of Health (NIH) Guide for the Care and Use of Laboratory Animals. The study is reported in accordance with Animal Research: Reporting of In Vivo Experiments (ARRIVE) guidelines. Ophthalmic examinations were performed according to a protocol approved by the University of California Davis Institutional Animal Care and Use Committee. Phenotypic data was collected from rhesus macaques previously evaluated for drusenoid lesions^21^. Some of the macaques in the present study were included in previous studies by Lin and coauthors^5^ (*n* = 67) and Casanova and colleagues (*n* = 52).^19^

The indoor macaques experienced a 12:12 light-dark cycle, with light levels in the facility varying from 70 to 930 lx depending on the area. The free-range macaques were housed in large outdoor enclosures with shaded and unshaded areas and thus exposed to light levels as high as 10,000 lx on a clear, sunny day. All macaques were transferred to the procedure room for evaluation, where light levels ranged from 700 to 1670 lx. The macaques were classified as “mostly indoor” or “mostly outdoor” if they spent more than 50% of their lifespan in either environment before their refractive error was measured. In addition, the same classification was recorded for the first 6 years of life to capture the initial phase of eye growth^11^. All measurements were obtained within a 6-hour period (8 a.m. to 2 p.m.), and no evaluation of diurnal variation in ocular parameters was conducted.

Comprehensive ophthalmic examination was performed on sedated macaques in the supine position under pharmacologic mydriasis and cycloplegia. Sedation was achieved by intramuscular injection of ketamine hydrochloride (5–30 mg/kg), dexmedetomidine (7.5–15 µg/kg) and/or midazolam (0.10 mg/kg). Topical tropicamide 1% (Akorn Inc., Lake Forest, IL, USA), phenylephrine 2.5% (Paragon BioTeck Inc., Portland, OR, USA) and cyclopentolate 2% (Alcon Laboratories Inc., Fort Worth, TX, USA) were instilled for mydriasis and cycloplegia. Macaques were monitored by a trained ophthalmic technician and veterinarian throughout the experiment.

### Refractive error

Objective cycloplegic refraction was performed by veterinarians with training in ophthalmology using streak retinoscopy and trial lenses in 0.25 D increments for sphere and cylinder, with a handheld retinoscope (Welch-Allyn Inc., New York, NY, USA). The power and axis of major and minor meridians were measured. Spherical equivalent refractive error was calculated; spherical equivalent refractive error = spherical power (diopters, D) + (cylinder power [D] / 2). Eyes were categorized based on their refractive error into groups defined by the American Optometric Association^22,23^. Eyes with a refractive error from ≥-0.50 to ≤ + 0.50 D were classified as emmetropic. Those with values between > + 0.50 D to ≤ + 2.00 D, >+2.00 D to ≤ + 5.00 D, and greater than + 5.00 D were categorized as low, moderate, and high hyperopia, respectively. Similarly, eyes with values between >-0.50 D to ≤-3.00 D, >-3.00 D to ≤-6.00 D, and <-6.00 D were grouped as low, moderate, and high myopia respectively. Any macaque with a refractive discrepancy of 1.00 D or more between their eyes was identified as having anisometropia.

Total astigmatism was defined as a difference in refractive power between the principal meridians (horizontal, vertical, or oblique) observed during streak retinoscopy^24,25^. Eyes were categorized based on their spherical equivalent refractive error, regardless of astigmatic magnitude or axis.

### Ocular biometry

Anterior segment tomography (Pentacam High-Resolution Tomographer, Oculus, Wetzlar, Germany) was performed to measure corneal curvature and anterior chamber (AC) properties, including ACV and ACD. Corneal astigmatism was calculated from corneal curvature measurements and it was further sub-classified based on the orientation of the principal meridians: with-the-rule (WTR) astigmatism, where the vertical meridian is steeper than the horizontal; against-the-rule (ATR) astigmatism, where the horizontal meridian is steeper than the vertical; and oblique astigmatism, where the steepest meridian lies between 30 and 60 degrees or 120–150 degrees^25^. Irregular astigmatism was defined as asymmetrical or non-orthogonal corneal curvature patterns not classifiable into WTR, ATR, or oblique axes.

A-scan ultrasound biometry (Sonomed Pacscan Plus, Escalon, Wayne, PA, USA) was performed to measure ACD (corneal endothelium to anterior capsule of crystalline lens), crystalline LT (anterior capsule to posterior capsule of crystalline lens), VCD (posterior capsule of crystalline lens to inner limiting membrane of retina) and AL (corneal epithelium to inner limiting membrane of retina) as previously described^4^. Specifically, a 10 MHz A-scan probe was perpendicularly placed over the central cornea with a coupling gel (Goniosoft, OcuSoft Inc., Richmond, TX, USA) following topical anesthesia with proparacaine (Bausch & Lomb, Tampa, FL, USA). Five A-scan recordings were obtained on the manual freeze mode when all the required echoes with sufficient height were present and averaged for each eye.

### Ophthalmic examination, including crystalline lens grading

All macaques underwent a comprehensive ophthalmic examination, including slit-lamp biomicroscopy, indirect ophthalmoscopy (Heine Optotechnik, Gilching, Germany) with a set of condensing lenses of different diopters and intraocular pressure measurement using rebound tonometry (TonoVet; Icare, Vantaa, Finland). Subjects with substantial posterior segment pathology were excluded from this study. Handheld slit lamp biomicroscopy was performed (SL-17, Kowa Optics, CA, USA) by a physician ophthalmologist and a veterinarian with expertise in ophthalmology. Crystalline lens changes including type, location and size of cataracts were documented using the semiquantitative preclinical ocular toxicology scoring (SPOTS) system;^26^ lenticular sclerosis was graded (nuclear sclerosis grade, NS-grade) using a clinically accepted (0–4) grading scale (**Supplementary Figure ****S1**), Lens Opacities Classification System II (LOCS II)^27^. A small number of macaques were assigned a clinical score of 1.5 to reflect lens opacities that fell between Grades 1 and 2 (**Supplementary Figure ****S1**). In addition, anterior segment tomography was performed using the Pentacam High-Resolution Tomographer (Oculus, Wetzlar, Germany) to measure the nuclear sclerosis density (Pentacam Nucleus Staging, NS-Pentacam) using a 0–5 grading scale (**Supplementary Figure S2**).

### Statistical analysis

Normality was determined by the Shapiro-Wilk test. Parametric data was presented as mean ± SD and nonparametric data as median and interquartile range (IQR). To calculate agreement between ACD measures with Pentacam vs. A-scan ultrasound and between, NS-grade vs. NS-Pentacam, a concordance correlation coefficient (CCC) and bias were calculated using values obtained from the same eye. For the CCC, the results were interpreted as previously described, with values of greater than + 0.75 indicating good agreement, values between + 0.40 and + 0.75 indicating moderate agreement, values of less than + 0.40 indicating poor agreement and negative values of the same magnitudes, indicating disagreement^19,28,29^. For normally distributed data, paired t-tests were used (ACD measures with Pentacam versus A-scan ultrasound values) while for comparisons with non-normal data (NS-Grade versus NS-Pentacam), Wilcoxon signed-rank test was used. Bland-Altman linear regression was used to evaluated agreement between anterior chamber depth measurements with Pentacam (ACD Pentacam) and A-scan ultrasound (ACD A-scan) and simple linear regression was used to investigate associations with the different parameters. Each rhesus macaque was treated as a random effect and all other variables were considered fixed effects. A Chi-square test was used to evaluate whether the indoor/outdoor environment across the entire lifespan influenced refractive error type (emmetropia, myopia, hyperopia). A *P* value of < 0.05 was considered statistically significant. R software (version 4.5.1; R Foundation for Statistical Computing, Vienna, Austria) was used for analyses requiring generalized estimating equations to account for inter-eye variability (GEE; geepack v1.3.12), correlation analyses, ordinal logistic regression, and Bland-Altman plots (ggplot2 v3.5.2). GraphPad Prism v9 (GraphPad Software Inc., La Jolla, CA, USA) was used for all remaining statistical analyses.

## Results

### Animals

Both eyes of 182 geriatric rhesus macaques from the CNPRC were evaluated with a mean age of 22.2 ± 2.5 (19.1–30.5) years. Geriatric status was defined as $\:\ge\:$19 years of age (human equivalence of approximately $\:\ge\:$57 years),^4^ with human age equivalence estimated at a 1:3 ratio^30,31^. Consistent with the demographics of the CNPRC breeding colony, 140 females and 42 males were included.

### Cycloplegic streak retinoscopy

**Fig. 1:**
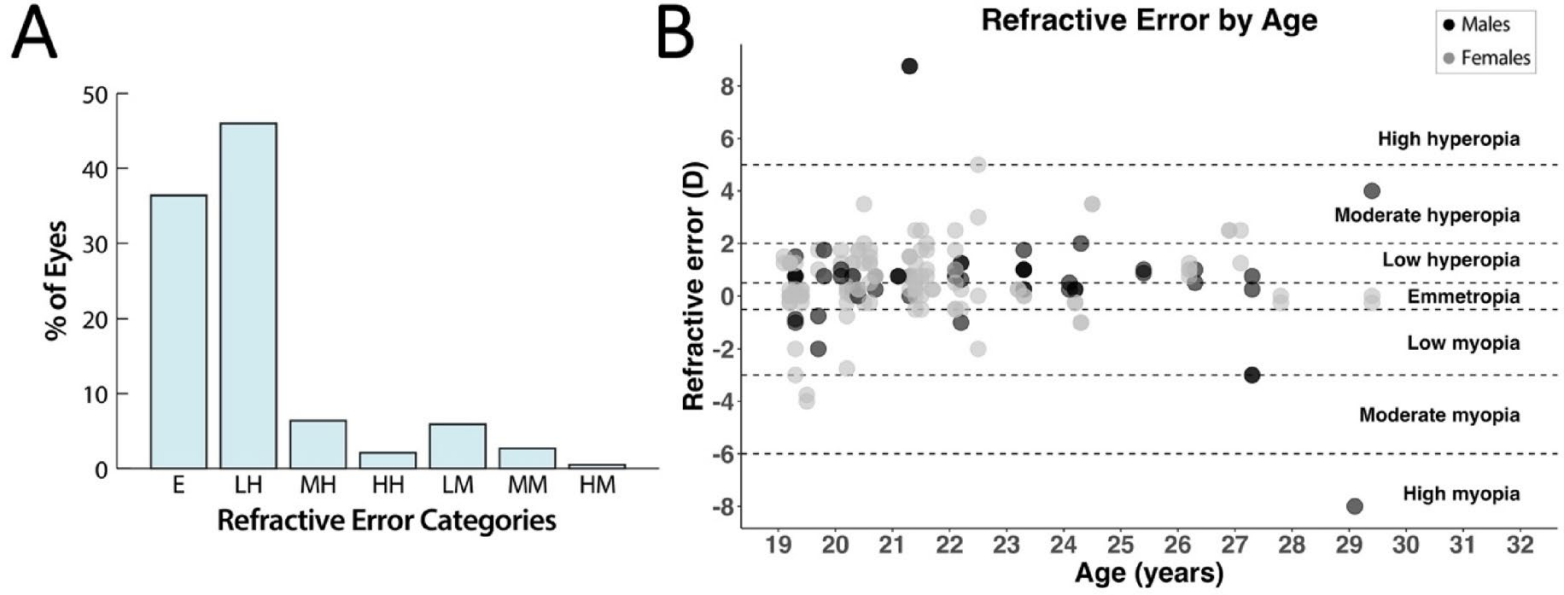
Low hyperopia and emmetropia were the most common refractive status observed in this population of geriatric rhesus macaques. (**A**) Retinoscopy was performed on 187 eyes of 95 animals (19–29 years of age); 55 were male and 131 were female. Spherical equivalent refractive error categories were defined as: emmetropia (E, ≥-0.50 to ≤ + 0.50 D, *n* = 68 eyes, 36%), low hyperopia (LH, >+0.50 to ≤ + 2.00 D, *n* = 86 eyes, 46%), moderate hyperopia (MH, > 2.00 to ≤ + 5.00 D, *n* = 12 eyes, 6%), high hyperopia (HH, >+5.00 D, *n* = 4 eyes, 2%), low myopia (LM, >-0.50 to ≤-3.00 D, *n* = 11 eyes, 6%), moderate myopia (MM, >-3.00 to ≤-6.00 D, *n* = 5 eyes, 3%), and high myopia (HM, >-6.00 D, *n* = 1 eye, 1%). (**B**) Refractive error was distributed across ages and sexes without a clear trend, with all the categories represented. Black dashed lines and right-aligned labels indicate refractive error categories based on spherical equivalent thresholds.

Objective cycloplegic streak retinoscopy was used to determine refractive error in 187 eyes of 95 rhesus macaques. Females were overrepresented in the population evaluated (28 males vs. 67 females), reflecting the colony proportions. The mean (± SD) refractive error spherical equivalence was + 0.7 ± 1.7 D. Total astigmatism was detected in 7 of the 187 eyes evaluated (3%). Of these, two eyes exhibited 1.50 D of total astigmatism, while the remaining five eyes showed ≤ 0.50 D. In 3 eyes, the presence of advanced cataracts prevented streak retinoscopy (**Supplementary Figure S3**). Most geriatric rhesus macaque eyes were hyperopic (*n* = 102, 55%) with emmetropic eyes also commonly observed (*n* = 68, 36%); myopes were least common (*n* = 17, 9%) (Fig. 1A). Both sexes were included in all the refractive error groups (Fig. 1B) and the refractive error was not significantly different in older macaques (*P* = 0.81). Anisometropia was observed in 13 rhesus macaques (14%).

Of the 95 rhesus macaques that underwent streak retinoscopy, only 3 (3%) were housed indoors before the age of 6 (**Supplementary Table ****S1**). Of these, 2 had hyperopia and 1 had myopia. Due to the small sample size, statistical analysis could not be performed for this group. However, when comparing the environmental conditions over the entire lifespan of the animals, no significant associations between the indoor/outdoor environment and refractive error were found (*P* = 0.53).

### Corneal curvature (Pentacam High-Resolution Tomographer) and anterior segment biometry (Pentacam and A-scan ultrasound)

A Pentacam High-Resolution Tomographer was used to measure corneal curvature and AC properties in 158 eyes of 91 rhesus macaques (75 females and 16 males). Corneal curvature was 52.6 ± 2.6 D (range: 43.0–61.8 D); ACV and ACD were 136 ± 17 mm^3^ (range: 102–180 mm^3^) and 3.34 ± 0.3 mm (range: 2.5–4.0 mm), respectively. Corneal astigmatism in our cohort differed by axis classification: ATR astigmatism averaged 1.44 ± 1.09 D at 21.3° ± 8.8° (*n* = 39, 25%); WTR astigmatism averaged 1.24 ± 1.07 D at 65.1 ± 6.2 degrees (*n* = 15, 9%); and oblique astigmatism averaged 1.57 ± 1.21 D at 42.6 ± 8.4 degrees (*n* = 98, 62%). Irregular astigmatism was identified in 1 of 158 eyes (1%), consistent with a very low prevalence in this geriatric macaque population. Ocular biometry measurements were also assessed via A-scan ultrasound biometry in 338 eyes of 171 rhesus macaques (134 females and 37 males). AL was 20.2 ± 1.5 mm (range: 17.5–28.7 mm), ACD was 3.7 ± 0.4 mm (range: 2.2–5.4 mm), LT was 4.1 ± 0.4 mm (range: 2.5–6.5 mm) and VCD was 12.2 ± 1.0 mm (range: 9.3–17.7 mm). Table 2 presents Pentacam keratometry values and A-scan measurements of anterior and posterior ocular segments across refractive error categories in geriatric rhesus macaques.

**Table 2:** Low hyperopia and emmetropia were the most common refractive errors observed in this population of geriatric rhesus macaques.

Refractive category	K (D)			Steep Axis (°)			Astig (D)			ACD (mm)			ACV (mm^3^)			LT (mm)			VCD (mm)			AL (mm)		
	Mean	SD	*n*	Mean	SD	*n*	Mean	SD	*n*	Mean	SD	*n*	Mean	SD	*n*	Mean	SD	*n*	Mean	SD	*n*	Mean	SD	*n*
Emmetropia	51.9	2.2	33	98.9	44.2	33	1.6	1.7	33	3.6	0.3	66	132.1	15.4	34	4.3	0.2	65	11.9	0.7	66	19.8	0.7	66
Low hyperopia	50.8	2.3	22	101.2	44.5	22	1.9	1.5	22	3.7	0.5	86	134.5	13.8	29	4.3	0.4	78	11.9	0.7	86	20	1.5	86
Moderate hyperopia	50.7	1	2	89.5	27	2	0.7	1	2	3.7	0.4	11	134.0	13.9	3	4.3	0.5	10	12.3	0.8	11	21	2.6	11
High hyperopia	52.7	1.6	3	97.3	58.2	3	1.7	0.6	3	3.7	0.1	4	122.5	7.8	2	3.7	0.5	3	11.9	0.1	4	18.6	2	4
Low myopia	47.7	3.6	5	142	17.4	5	2.2	1.8	5	4	0.6	11	153.4	20.1	5	4.1	0.3	10	12.8	0.7	11	20.8	0.9	11
Moderate myopia	N/A			N/A			N/A			3.7	0.4	5	112.5	3.5	2	4.2	0.3	5	12.2	0.4	5	20.1	0.3	5
High myopia	N/A			N/A			N/A			*2.9	N/A	1	N/A			5.0	N/A	1	*16.1	N/A	1	*22.0	N/A	1

The table includes values of keratometry acquired via Pentacam and A-scan measurements of anterior and posterior ocular segment among refractive error categories in geriatric rhesus macaques. The table included following ocular biometry values acquired with Pentacam: K – average keratometry reading measured in diopters (D), Steep Axis – the average location of the steepest meridian in degrees, Astig – average corneal astigmatism measured in diopters (D), and values acquired with the A-scan: ACD - anterior chamber depth measured in millimeters (mm), ACV - anterior chamber volume (mm^3^), LT – lens thickness (mm), VCD – vitreous chamber depth measured in millimeters (mm), and AL – axial length measured in millimeters (mm). Note – Pentacam values were not available for moderately and highly myopic animals, labeled N/A. In the high myopia group there was only one eye available for evaluation with A-scan, because of this the mean value is equivalent to the measurement in the single eye (marked with asterisk *). n indicates the number of eyes evaluated in each refractive category.

#### Differences in ACD measured via Pentacam tomography versus A-scan ultrasound

**Fig. 2:**
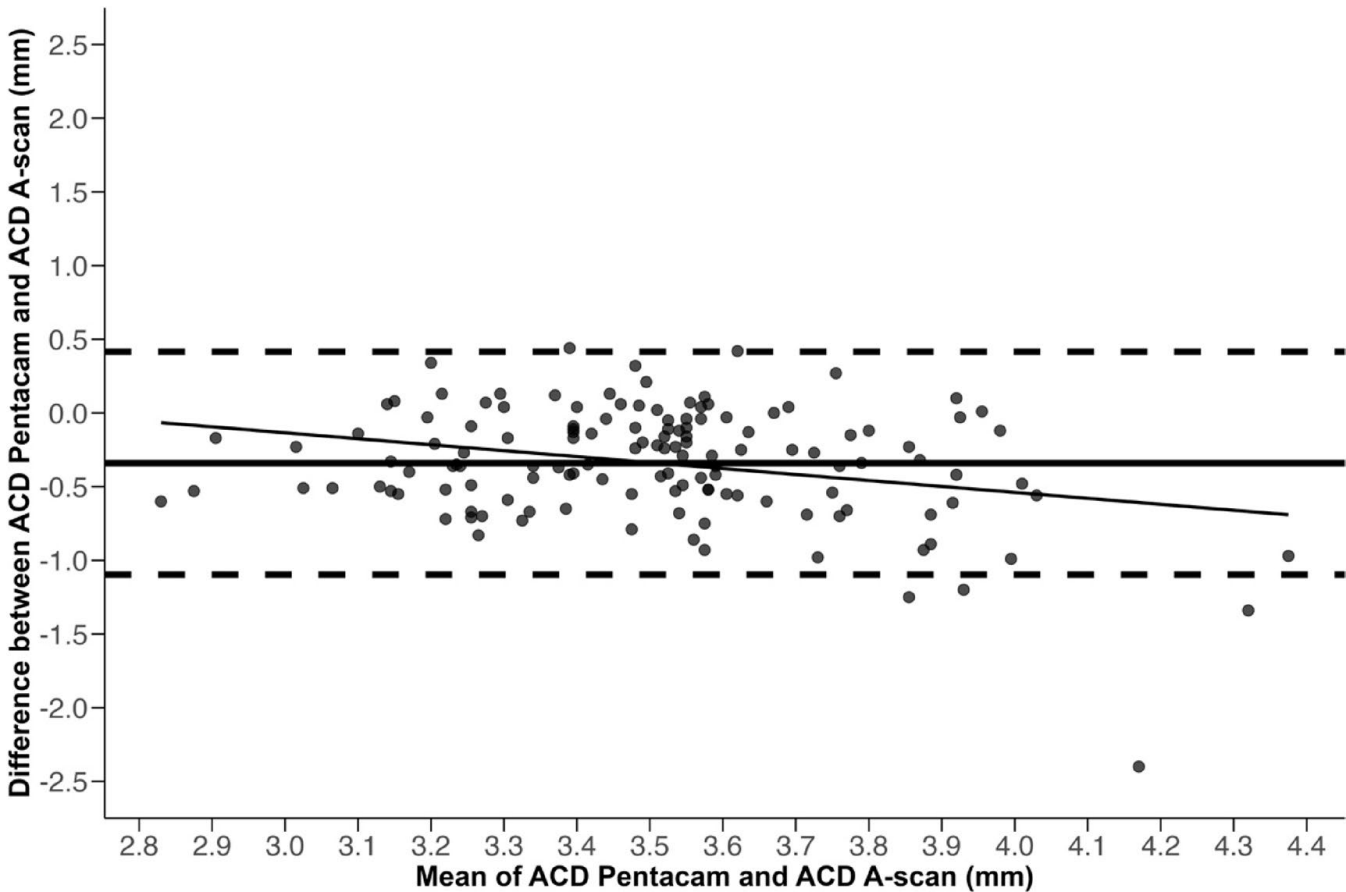
Bland-Altman plot indicates that Pentacam underestimates the anterior chamber depth (ACD) at higher values and overestimates it at lower values when compared to A-scan. The anterior chamber depth was measured with Pentacam and A-scan ultrasound in 136 eyes. The vertical axis represents the difference between the two types of measurements and the horizontal axis plots the mean value for the two types of measurements. The dashed lines represent the 95% limits of agreement, and the black line represents a negative slope (*P* < 0.001). The concordance correlation coefficient (CCC) was 0.2 with a confident interval of 0.1–0.3, indicating poor agreement between the two measurement techniques. All data points are grey with transparency; overlapping points may appear darker due to layering.

Differences in ACD measured via tomography (ACD Pentacam) versus A-scan ultrasound (ACD A-scan) are represented in Fig. 2. Bias of ACD Pentacam versus ACD A-scan was equal at -0.3 and a significantly negative slope (*P* < 0.001) was identified indicating that Pentacam underestimates ACD at higher values and overestimates it at lower values when compared to ultrasound A-scan measurements. The CCC of 0.2 demonstrated poor agreement between the two techniques and a Wilcoxon test demonstrated a statistically significant difference in ACD between the two measurements (Pentacam = 3.4 ± 0.3 mm; A-scan = 3.7 ± 0.4 mm, *P* < 0.001).

### Slit lamp biomicroscopy and Pentacam findings in the lens

**Fig. 3:**
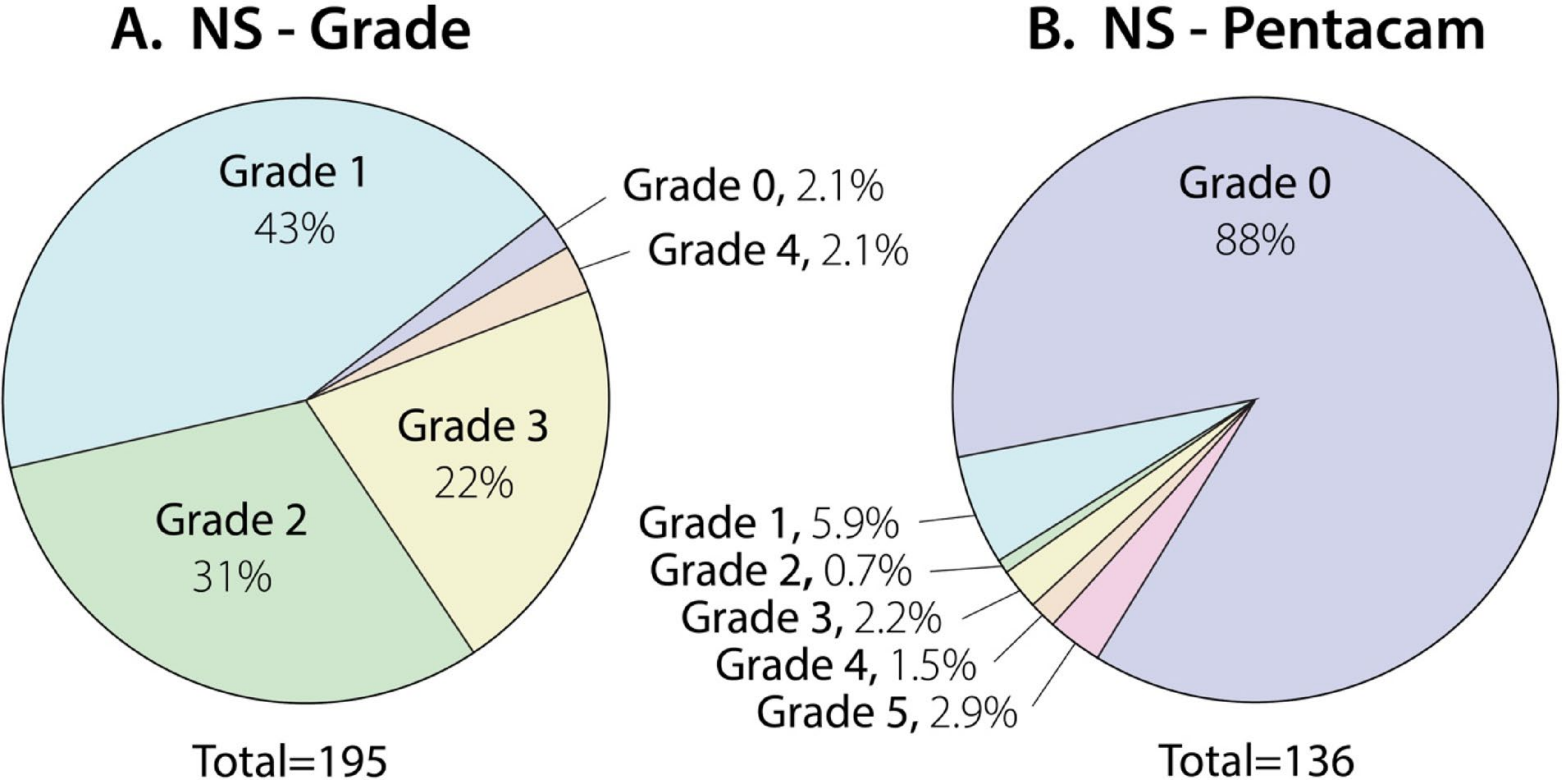
There was poor agreement between nuclear sclerosis grade determined by examiners (NS-Grade) and nucleus staging determined based on Pentacam internal algorithm (NS-Pentacam). The two pie charts demonstrate percentage of eyes affected by various grades of nuclear sclerosis and most commonly observed lens opacities within the evaluated population of geriatric rhesus macaques. NS-Grade (**A**) represents the degree of nuclear sclerosis, as determined by a physician ophthalmologist or a veterinarian with expertise in ophthalmology in 195 eyes of 99 geriatric rhesus macaques (Lens opacities classification system II—LOCS II: 0–4 scale); while a small number of eyes were clinically graded as 1.5, these were rounded to Grade 2 for clarity in this figure. NS-Pentacam (**B**) represents the Pentacam Nucleus Staging, as calculated by anterior segment tomography via the Pentacam in 136 eyes (0–5 scale). Due to rounding of percentages, the cumulative percentage for each figure may appear be > 100%.

**Fig. 4:**
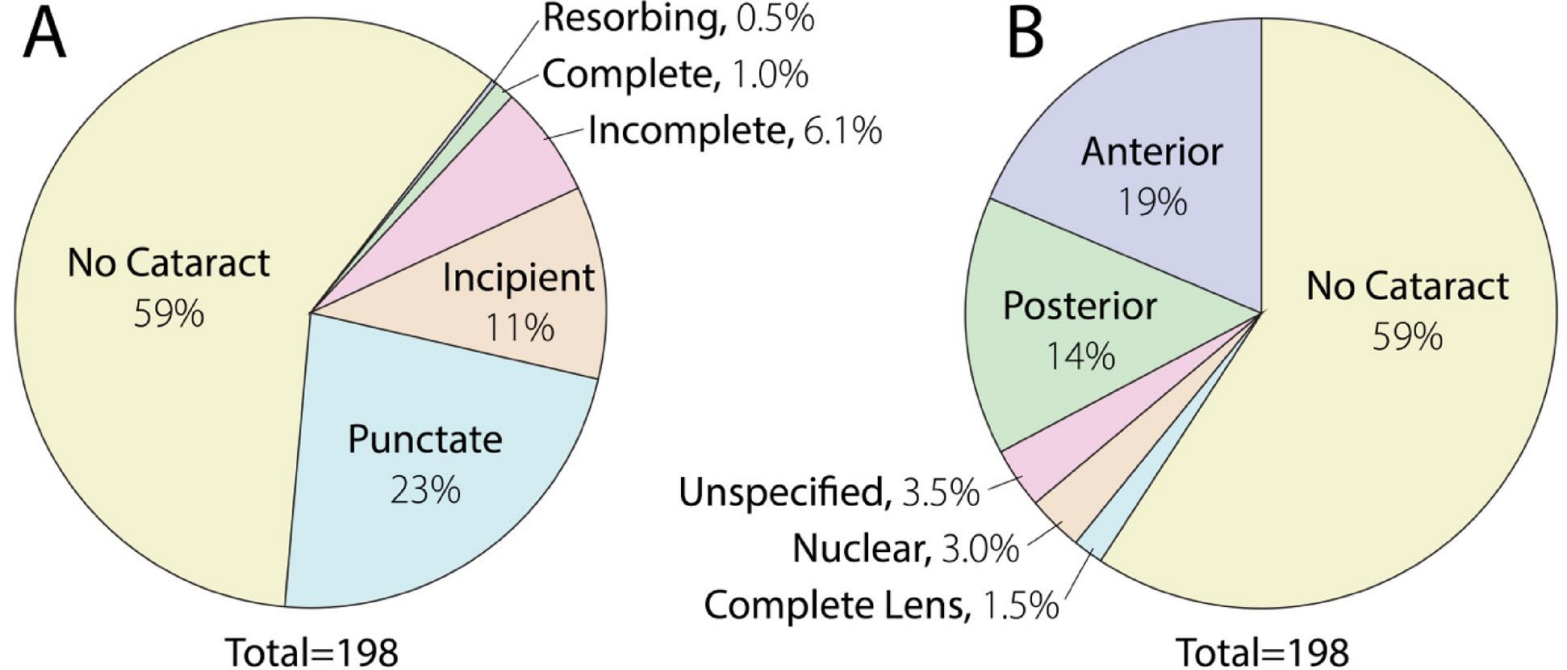
Cataract was observed in 41% of geriatric rhesus macaque eyes. Cataract severity (**A**) was graded as incipient, punctate, incomplete, complete, resorbing while locations (**B**) included posterior opacity, anterior opacity, nuclear, unspecified location or complete lens. The most frequently observed severity of cataracts was punctate (*n* = 45) and predominantly localized anteriorly (*n* = 37) or posteriorly (*n* = 28). Only a few eyes had complete (*n* = 2) or resorbing (*n* = 1) cataracts. Location and size of cataracts were documented using semiquantitative preclinical ocular toxicology scoring (SPOTS) system. Due to rounding of percentages the cumulative percentage for each figure may be > 100%.

With slit lamp biomicroscopy, 191 of 195 (98%) eyes of 99 macaques had nuclear sclerosis. Median (IQR) NS-grade was 1 (1–2) and 47 eyes (24%) had a grade > 2 (Fig. 3A). Four eyes had no nuclear sclerosis (grade 0), two eyes had a complete cataract, and one eye had a resorbing cataract **(**Fig. 4A**)**. Punctate cataracts were most frequently observed (45 of 198 eyes, 23%) followed by incipient and incomplete cataracts (22 and 12 of 198 eyes, 11% vs. 6%). Cataracts were most frequently located in the anterior and posterior cortex (37 and 28 of 198 eyes, 19% vs. 14%) **(**Fig. 4B**).** Median NS-Pentacam, as calculated by anterior segment tomography via the Pentacam in 136 eyes of 75 primates was 0 with an IQR of 0 to 0, with only 18 eyes having score > 0 (13%).

Nuclear sclerosis grade vs. NS-Pentacam were compared in Fig. 3A-B. Although the NS-grade (0–4 subjective scale) and NS-Pentacam (0–5 objective scale) differ in scoring criteria, we compared them to evaluate how well the objective Pentacam-based measure corresponds with standard clinical grading, providing insight into the relative utility of each method for assessing nuclear sclerosis. Bias of NS-grade versus NS-Pentacam was identified at 1.4 with a significant negative slope (*P* = 0.3) and poor correlation (CCC = 0.2); a Wilcoxon test confirmed statistically significant differences between the two measurement types (*P* < 0.001) suggesting that the Pentacam Nucleus Staging significantly underestimates the degree of nuclear sclerosis when compared to the gold standard slit lamp grading of nuclear sclerosis.

As expected, a significant increase in NS-Grade and NS-Pentacam scores with age (19.1–30.5 years) was observed using ordinal logistic regression (*P* < 0.001 for both). We observed a significant positive correlation between slit-lamp nuclear sclerosis scores and the hyperopic group (*P* = 0.0012; **Supplementary Figure S4**). No significant associations were observed between nuclear sclerosis severity and refractive error in the emmetropic or myopic groups, regardless of the assessment method (**Supplementary Figure S4**).

### Refractive status and ocular biometric measurements

**Fig. 5:**
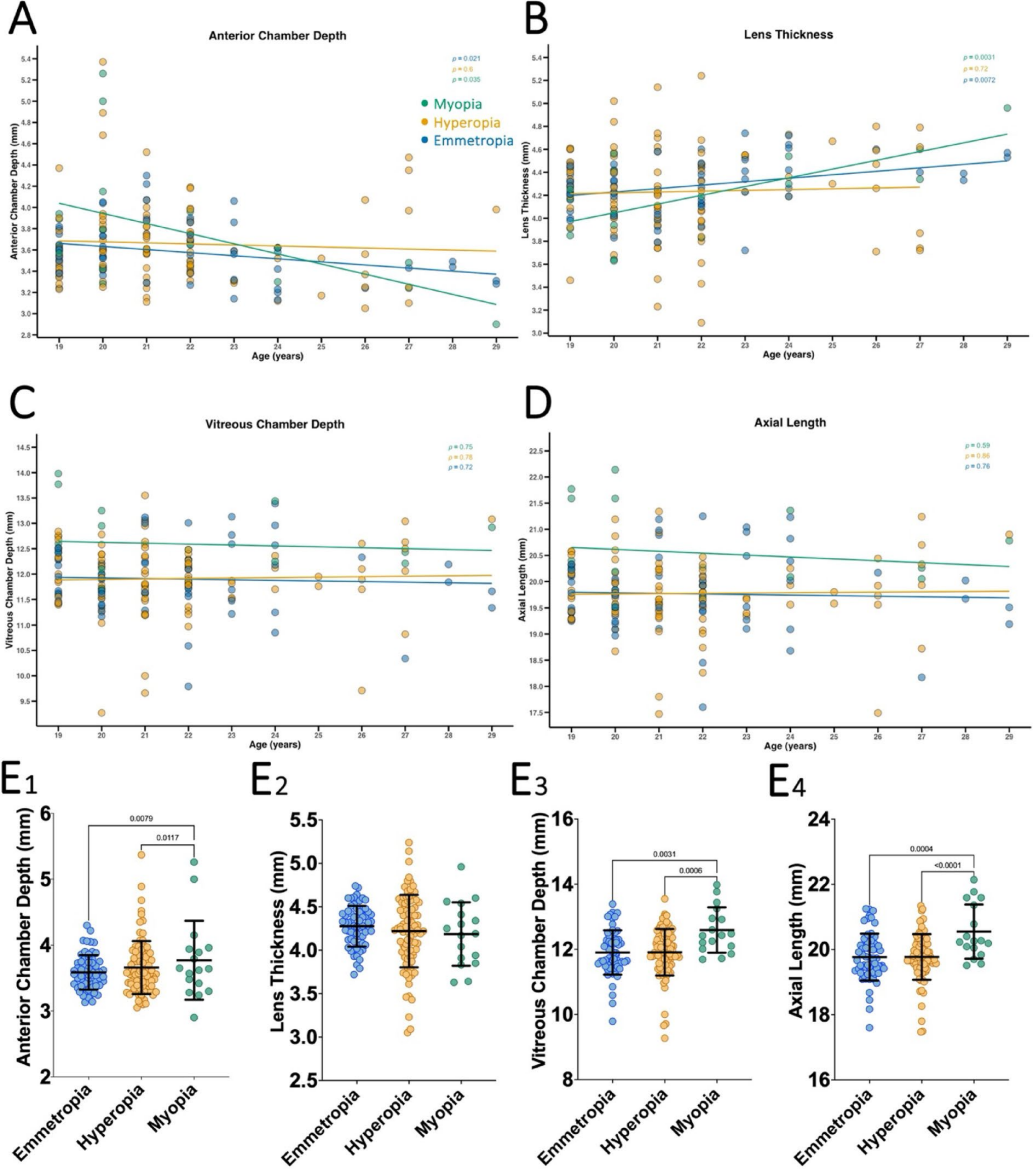
Age-related and refractive group differences in ocular biometry parameters measured by A-scan in geriatric rhesus macaques. Panels (**A**–**D**) show anterior chamber depth (ACD; **A**), lens thickness (LT; **B**), vitreous chamber depth (VCD; **C**), and axial length (AL; **D**) as a function of age, stratified by refractive error group. Panels (**E1**–**E4**) present group-wise comparisons of ACD (**E1**), LT (**E2**), VCD (**E3**), and AL (**E4**) across hyperopic, emmetropic, and myopic eyes. For panels (**E1**–**E4**), group data are presented as mean ± standard deviation (SD). *P*-values from statistical comparisons are indicated.

Biometric parameters from A-scan were analyzed across the different refractive error categories to gain further insights into the underlying causes of these refractive states. We observed that the ACD was significantly shallower in older myopes and emmetropes (*P* < 0.05 in both; Fig. 5A), likely due to age-related lens thickening in these groups (*P* < 0.01 in both; Fig. 5B). By contrast, VCD (Fig. 5C) and AL (Fig. 5D) did not significantly change across all refractive error groups in older macaques, consistent with the expectation that globe growth has ceased at this stage and no macaque had increased IOP that could lead to buphthalmia. Myopes had significantly deeper ACD (3.77 ± 0.59) when compared to emmetropes (3.59 ± 0.26; *P* < 0.01) and hyperopes (3.66 ± 0.40; *P* < 0.05, Fig. 5E1). There were no significant differences between LT between the refractive error groups (Fig. 5E2). Myopes also had significantly deeper VCD (12.59 ± 0.69) when compared to emmetropes (11.90 ± 0.68; *P* < 0.01) and hyperopes (11.91 ± 0.71, *P* = 0.001; Fig. 5E3). Similarly, myopes had significantly longer AL (20.55 ± 0.83) when compared to emmetropes (19.77 ± 0.72; *P* < 0.001) and hyperopes (19.77 ± 0.69, *P* < 0.001; Fig. 5E4).

Additionally, we explored whether the corneal biometric parameters measured by Pentacam differed between refractive error groups by age, to further characterize anterior segment differences in geriatric rhesus macaques. Mean keratometry (**Supplementary Figure S5A**) showed a non-significant trend toward flatter corneas with increasing age in emmetropic eyes (*P* = 0.07). Steep axis orientation (**Supplementary Figure S5B**) exhibited a trend toward higher axis values with age in these same group (*P* = 0.11), indicating a more vertical steep meridian. Corneal astigmatism (**Supplementary Figure S5C**) remained relatively stable across the age range, with a slight decrease in myopic and emmetropic eyes and a mild increase in hyperopic eyes, none of which were statistically significant. Additionally, no significant group differences were detected for these parameters (**Supplementary Figure S5A2-C2)**.

## Discussion

Herein, we report refractive error, ocular biometry characteristics and age-related lens changes in a large geriatric rhesus macaque population at the CNPRC. While refractive error has been previously reported in immature and adult rhesus macaques,^32,33^ our study measured refractive error and lens changes in a large population of rhesus macaques ≥ 19 years of age. We determined that low hyperopia and emmetropia were the most common refractive states in this geriatric population consistent with previously shown hyperopic emmetropization in adolescent macaques with an average refractive value of + 2.00 D.^34^ In the present study, myopia was less common (*n* = 17, 9%) in geriatric rhesus macaques with high myopia being the least common refractive error (*n* = 1, 1%). Indeed, Tzu-Ni Sin and colleagues described an NHP model of myopic foveoschisis amongst a cohort of 40 myopic rhesus macaques with AL > 21 mm (age 7.3–29.0 years old) at the CNPRC^35^. We note that cycloplegic retinoscopy may overestimate the degree of hyperopia in this population versus psychophysical methods^36^.

Nevertheless, the predominance of low refractive error is expected in rhesus macaques given their lifestyle. Limited near activity and outdoor living likely reduce the stimulus for myopia development and progression and subsequently also reduces the risk of myopia-related ocular health complications, including retinal detachment^37–40^. While there is no direct evidence linking indoor housing to refractive errors in rhesus macaques, existing studies suggest that environmental factors, particularly lighting conditions, may influence refractive error development^41^. Exposure to natural outdoor light has been shown to reduce the risk of myopia in rhesus macaques, with those housed under dim light conditions more likely to develop refractive errors^42,43^. Therefore, in our study, the low number of animals housed indoor in the first six years of life (3%) likely contributed to the generally low prevalence of refractive errors observed in our colony. Given the low prevalence of myopia in rhesus macaques, further understanding of the mechanisms that lead to this may provide valuable insights into the underlying factors causing the development and progression of nearsightedness in human patients. Further studies involving a broader sample size and varying housing conditions will be crucial in understanding how early environmental factors, including light exposure, affect refractive error development in rhesus macaques. Interestingly, the much lower prevalence of astigmatism observed in our monkey cohort (3%) compared to geriatric human populations (83%) highlights notable species differences in corneal structure and refractive development^44^.

In the present study, anisometropia (≥ 1.00 D) was present in 14% of the geriatric rhesus macaques that were refracted. The prevalence of anisometropia in the human population varies widely depending on inclusion criteria, but can generally be estimated at ~ 19%.^45^ In the current study, a notable proportion of rhesus macaques had a degree of anisometropia that if present in early life could have put them at potential risk for amblyopia. However, the purview of this study was limited, and we were unable to determine if this amblyogenic anisometropia was present early in life among our population.The best estimate of amblyopia among humans is 2% with anisometropic and strabismic amblyopia accounting for 90% of all amblyopic cases^46,47^. Interestingly, no rhesus macaques included in this investigation demonstrated frank signs of large angle strabismus during clinical examination, whereas strabismus in the human population is estimated to be between 2–5%.^48^ The presence of darker scleral melanin in rhesus macaques may create more challenges in diagnosing strabismus in this species and anisometropia may be the more common risk factor for amblyopia in the rhesus macaque population. A future survey of spontaneous anisometropia and possible amblyopia among normal young rhesus macaques will be important for understanding the mechanism of disease in humans. In addition, the seemingly low prevalence of strabismus in this population warrants further investigation. Furthermore, it will be important to assess amblyopia, with a particular focus on evaluating the role of high bilateral isometropia in the development of amblyopia in this animal model. The quality of life in geriatric primates with amblyopia may further decrease as they develop presbyopia, leading to discomfort and visual impairment. Unfortunately, presbyopia was not evaluated in current study, but it would be an important future investigation topic in geriatric rhesus macaques.

Corneal tomography confirmed that rhesus macaques have significantly steeper mean corneal curvature (52.6 ± 2.6 D) versus humans (43.8 ± 1.4 D).^49^ The ATR corneal astigmatism is known to increase with age in humans^50^. Our study confirmed an elevated prevalence of oblique (62%) and ATR (24%) corneal astigmatism in this geriatric rhesus macaque population. Subjects with corneal astigmatism > 2.00 D was approximately 30% and > 3.00 D was approximately 15%. Conversely, the low presence of irregular astigmatism (1%), despite steep mean corneal curvature, and minimal corneal pathological findings during anterior segment evaluation is suggestive of a low frequency of corneal dystrophy, including keratoconus, in this population. Future studies should utilize corneal topography to better understand the prevalence of anterior and posterior corneal irregularity in this species to confirm its potential as a model for elucidating the mechanisms associated with corneal dystrophies.

Anterior and posterior biometry measurements in geriatric rhesus macaques are similar to those of geriatric humans^51,52^. As previously reported by our group, in our study ACD (3.7 ± 0.4 mm versus 2.5, range: 2.5–2.5 mm^51^, respectively) was greater while LT (4.1 ± 0.4 mm versus 4.5, range: 4.4–4.5 mm,^51^ respectively) and VCD (12.2 ± 1.0 mm versus 15.6, range: 15.5–15.6,^51^ respectively) was lower in geriatric rhesus macaques compared to humans^51,52^. Relative to AL, the ratio of ACD: LT: VCD is ~ 1:1:3 in geriatric rhesus macaques and ~ 1:2:6 in humans 60–64 years of age^51,53^. Our study also confirmed poor agreement between ACD measurements obtained with two different methods, A-scan ultrasound and the Pentacam High-Resolution Tomographer. Future studies utilizing the ACD measurement should consider the variability of this measure between instruments during study design. While the Pentacam provides reproducible data and has been applied successfully in other species (e.g., dogs^54^, its use in rhesus macaques has not yet been formally validated against a gold standard technique. Therefore, without cross-comparison to established reference methods, the anatomical accuracy of Pentacam-derived measurements remains uncertain. Future investigations should aim to validate the Pentacam in rhesus macaques to ensure both precision and accuracy of biometric parameters.

Although previous studies, including those from our group and others, have reported ocular biometric measurements in rhesus macaques, this study is the first to categorize these parameters by refractive error^5,34^. This approach offers a more comprehensive understanding of the relationship between biometric alterations and refractive changes, thereby advancing the characterization of aging in rhesus macaques. In our rhesus macaque population, we confirmed that myopic individuals exhibit significantly longer ACD, VCD and AL than emmetropes and hyperopes, consistent with an axial component to their refractive error. In all refractive error groups, LT was higher in older individuals, as it has been described to occur with age in this species^5,34^. However, we did not observe statistically significant differences in biometric parameters between hyperopic and emmetropic individuals. Based on these findings, we hypothesize that the nature of refractive error in this animal model is mixed, primarily axial in myopes, whereas hyperopia may be driven by refractive components, as their biometric measurements alone do not fully account for the observed refractive error. It is important to note that determining the precise nature of hyperopia was beyond the scope of this study, and future investigations should consider collecting additional data to further elucidate the underlying mechanisms. Although not statistically significant, trends in Pentacam-derived corneal parameters suggest subtle, age-related changes. Keratometry (K) showed slight flattening with age, consistent with age-related corneal flattening observed in humans^55,56^. The tendency toward a more vertical steep axis in may reflect biomechanical shifts or eyelid tension changes with age. These modest changes align with the generally stable corneal profile reported in aging human eyes^55^.

In this study, 24% of rhesus macaque eyes had nuclear sclerosis that would visually impair humans (≥ 3 grade); 2% of eyes assessed had grade 4 nuclear sclerosis. In our population, we observed a lower occurrence of cataract than anticipated (41%) with the most frequently observed type being punctate cataracts (23%) localized to the anterior (19%) and/or posterior cortex (14%). With aging, humans experience more frequent anterior and posterior lenticular changes^57^. The lower-than-expected occurrence of cataracts in our study subjects contrasts with the high prevalence observed in humans, where 73% of females and 78% of males aged 65 to 74 develop cataract^58^. We also determined that the extent of nuclear sclerosis formation (as determined by an ophthalmologist using a clinically accepted 0–4 grading scale) should be considered independently from the NS-Pentacam, a measure of crystalline lens density calculated by the Pentacam High-Resolution Tomographer on 0–5 scale. These methods showed poor agreement and clinical grading should be prioritized as the reference standard for nuclear sclerosis assessment, as the latter is subject to greater variability due to opacity location, density and image alignment. Previous studies have demonstrated the correlation between nuclear sclerosis progression and ultraviolet (UV) light exposure^59^. Thus, this relatively low prevalence of advanced nuclear sclerosis in our population of rhesus macaques was unexpected, given that they spend a majority of their life outdoors. Additionally, nuclear sclerosis severity did not correlate with age, suggesting limited progression or inter-individual variability in late life. This suggests that other physiological properties, such as corneal and lens UV light absorption, may differ in rhesus macaques versus humans or that other unknown environmental factors may be involved. A significant association between nuclear sclerosis severity and hyperopia was observed, though limited to slit-lamp grading and not seen in other refractive groups or assessment methods. This pattern is compatible with age-related lenticular changes accentuating pre-existing hyperopia, at least at the relatively mild to moderate nuclear sclerosis stages captured here. In humans, longitudinal population-based studies have shown that refraction often shifts toward hyperopia with early age-related reductions in crystalline lens power, whereas more advanced nuclear cataract is associated with a later myopic shift^60^. However, hyperopic eyes may also be structurally predisposed to nuclear lens change, and our cross-sectional data cannot determine whether hyperopia is a cause or a consequence of increasing nuclear sclerosis. Although the study population was geriatric, total years of life expectancy are greater in humans versus rhesus macaques and could account for the difference in the extent of cataract present at this stage. Further investigation into the multifactorial properties associated with cataract formation is needed for elucidating the underlying mechanisms.

This study is not without limitations, most of which are due to the extensive nature of the evaluation, which included multiple tests and parameters collected over several years by different evaluators. One limitation is that not all macaques could be evaluated with all techniques, resulting in some missing data. Another limitation is the use of the American Optometric Association refractive classifications, which are based on human standards and may not fully reflect the unique characteristics of rhesus macaques. Additionally, the AOA classifications are based on subjective refraction, whereas our study employed cycloplegic objective refraction methods for measuring refractive error in rhesus macaques. Therefore, we acknowledge that applying human-based classification criteria to NHP models, combined with the methodological differences in refraction techniques, may result in potential discrepancies. Additionally, we did not evaluate diurnal variation in ocular parameters, which may influence certain biometric measurements and should be considered in future studies. Given the high interest in studying cognition and neurodegeneration in geriatric rhesus macaques,^61^ it is imperative that ocular health be assessed as many behavioral tasks require normal vision to complete. This study gives important insights into the prevalence of lens changes and refractive error that could impact vision and quality of life in geriatric rhesus macaques. Furthermore, identification of these abnormalities may require provision of environmental enrichment or accommodations to preserve normal behavior and social order^62,63^.

## Conclusion

Hyperopia is the most prevalent refractive error in the geriatric rhesus macaque population at the CNPRC. Further understanding of the mechanisms that lead to low prevalence of myopia, as observed in current study, may provide valuable insights into the underlying factors causing the development and progression of nearsightedness in human patients. Finally, the low occurrence of vision decreasing crystalline lens changes could be attributed to the difference in the lifespan between rhesus macaques and humans and create an opportunity for additional studies regarding cataract formation and treatment.

## Supplementary Information

Below is the link to the electronic supplementary material.

Supplementary Material 1

## Data Availability

The data that support the findings of this study are available on request from the corresponding author, Sara M. Thomasy. The data are not publicly available.

## Abbreviations

AC: Anterior chamber
ACD: Anterior chamber depth
ACV: Anterior chamber volume
AL: Axial length
Astig: Corneal astigmatism
ATR: against-the-rule
CCC: Concordance correlation coefficient
CNPRC: California National Primate Research Center
D: Diopters
IQR: Interquartile range
K: Average keratometry reading measured in diopters with Pentacam
LOCS II: Lens opacities classification system II
LT: Lens thickness
NHPs: Non-human primates
NS-Grade: Nuclear sclerosis grade
NS-Pentacam: Pentacam nucleus staging
SD: Standard deviation
SPOTS: Semiquantitative preclinical ocular toxicology scoring system
VCD: Vitreous chamber depth
WTR: With-the-rule
