# Supplementary material for: Spontaneous refractive error, ocular biometry and age related lens changes in a population of geriatric rhesus macaques
Source: Sci Rep. 2025 Dec 5;16:962. doi: 10.1038/s41598-025-30581-6 (PMC12783095; doi:10.1038/s41598-025-30581-6)
Supplement: Supplementary file 1 — Supplementary Material 1 [file 41598_2025_30581_MOESM1_ESM.docx]

# Supplementary Figure S1

**
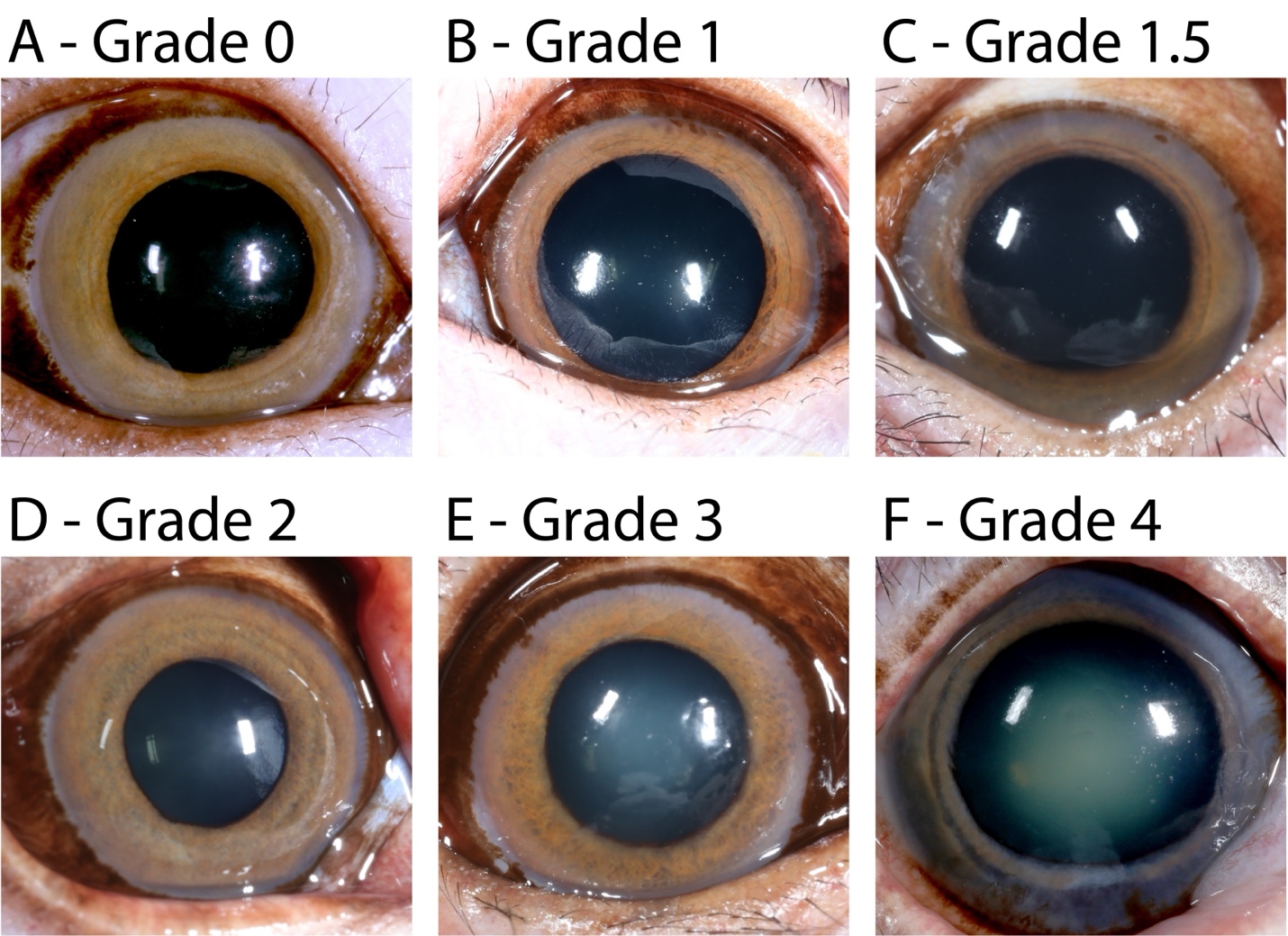
**

**Supplementary Figure S1. Evaluation of nuclear sclerosis with slit lamp biomicroscopy enables the identification of 6 different clinical grades in geriatric rhesus macaques. A,** Grade 0, normal lens with no opacity detectable. **B,** Grade 1 - Minimal opacity; the nucleus is clear with no noticeable cloudiness. **C**, Grade of 1.5 represents a mild level of lens nucleus cloudiness that falls between Grade 1 (clear/minimal opacity) and Grade 2 (mild opacity). **D,** Grade 2 - Mild opalescence; the nucleus is slightly cloudy but not significantly impairing vision. **E,** Grade 3 - Moderate opalescence; cloudiness is visible and may affect visual acuity. **F,** Grade 4 - Severe opalescence; the nucleus is highly opaque, significantly impairing vision.

# Supplementary Figure S2

**
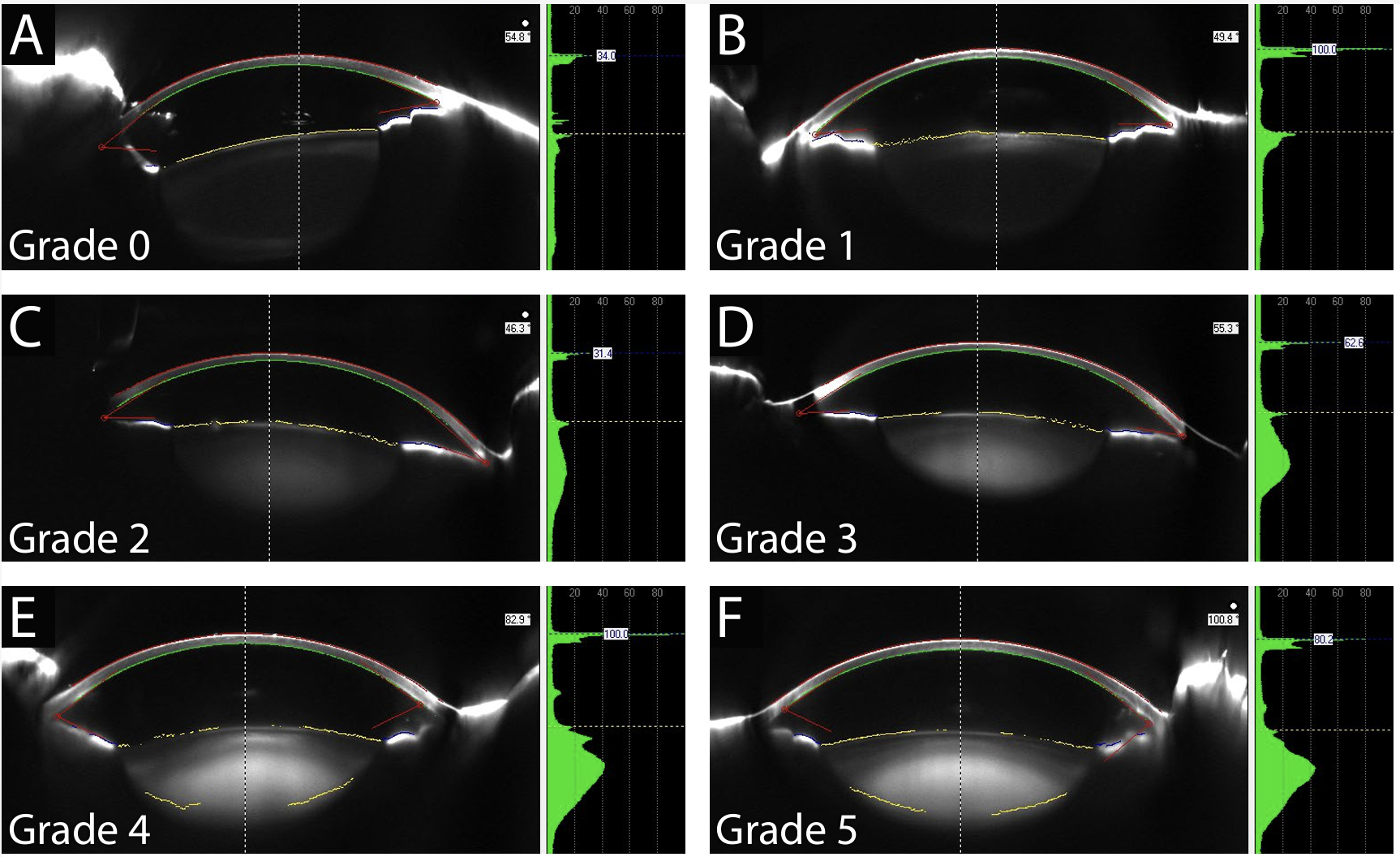
Supplementary Figure S2. Evaluation of nuclear sclerosis with Pentacam enables the identification of 6 different clinical grades in geriatric rhesus macaques, including grade 0 as the normal reference. A**, Grade 0 - Clear nucleus with no visible sclerosis. **B**, Grade 1 - Very mild sclerosis with minimal lens opacity. **C**, Grade 2 - Mild sclerosis with slight increase in light scatter but still good transparency. **D**, Grade 3 - Moderate sclerosis; noticeable opacity with significant impact on transparency and light scatter. **E**, Grade 4 - Severe nuclear sclerosis; pronounced opacity with high density. **F**, Grade 5 - Extremely dense nucleus, often appearing as an advanced "brunescent" cataract.

# Supplementary Figure S3


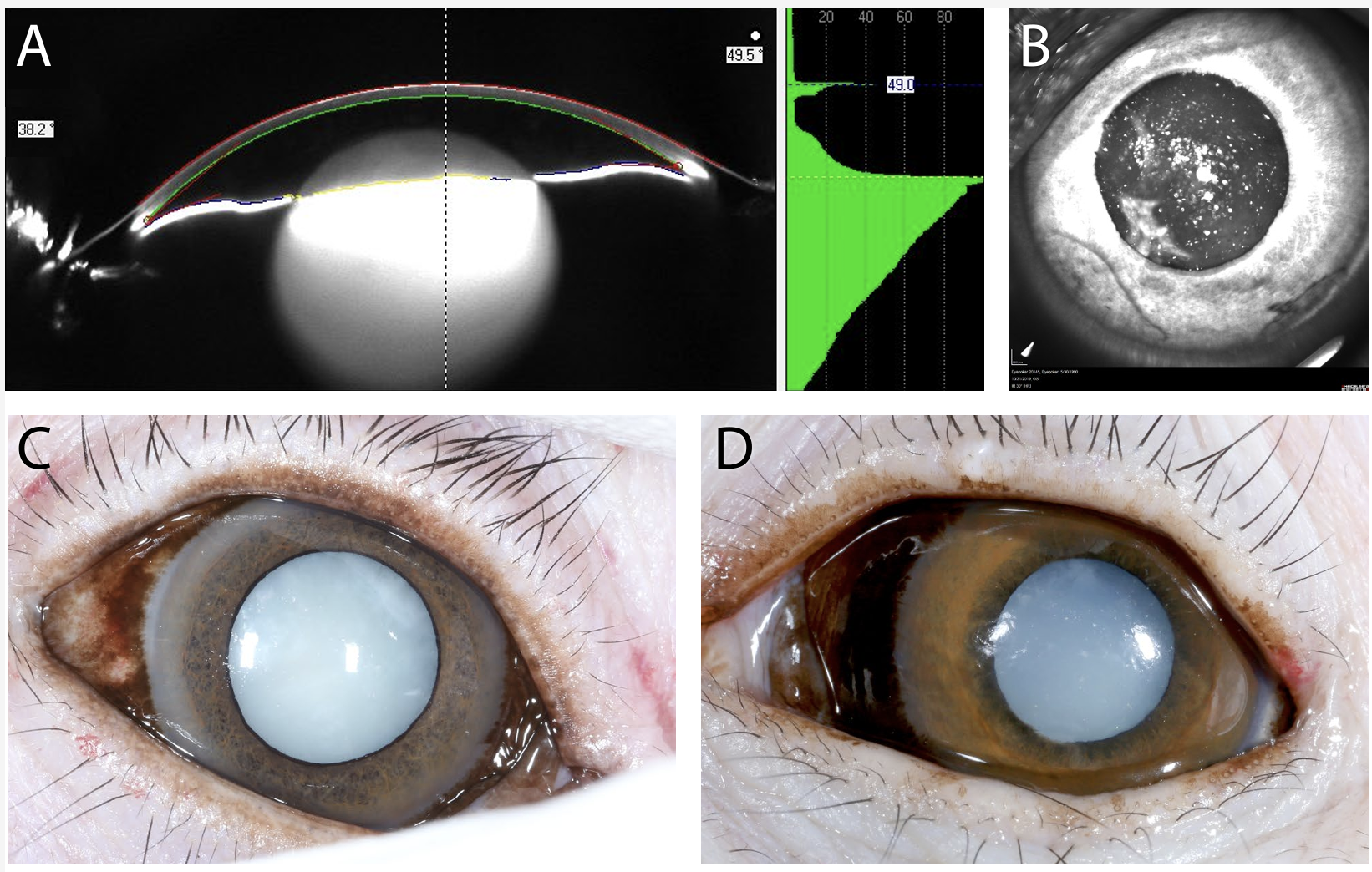


**Supplementary Figure S3. Multimodal evaluation of advanced cataract in a representative rhesus macaque illustrating the inability to perform streak retinoscopy due to media opacity. A,** Pentacam anterior segment image. **B,** Infrared confocal scanning laser ophthalmoscopy (cSLO) image showing lack of retinal reflex through the pupil. **C-D,** Two examples of representative animals in which the advanced cataract completely blocks the retinal reflex.

# Supplementary Figure S4


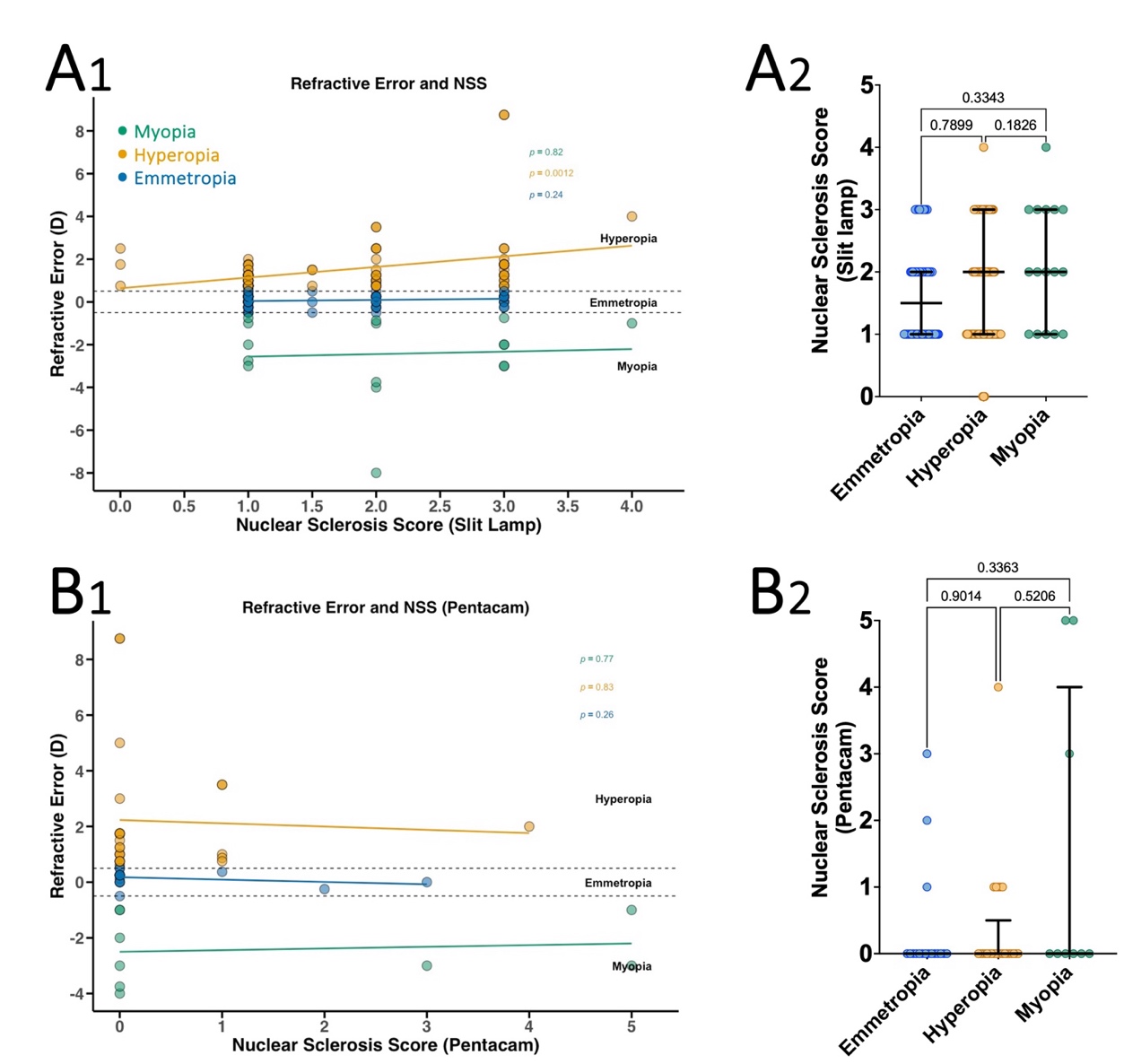


**Supplementary Figure S4. Association between refractive error and nuclear sclerosis severity in geriatric rhesus macaques. A1**, Scatterplot of refractive error (D) as a function of nuclear sclerosis score (NSS) measured by slit-lamp biomicroscopy, stratified by refractive error group (myopia, hyperopia, and emmetropia). **A2**, The corresponding group-wise comparison of NSS values across refractive error groups; no significant differences were observed. **B1**, Same scatterplot using NSS measured by Pentacam. **B2**, Group-wise comparison of Pentacam-derived NSS across refractive error groups. For panels **A2** and **B2**, boxplots display the median and interquartile range (IQR). No significant correlations or group differences were detected in any panel. *P*-values from statistical comparisons are indicated.

# Supplementary Figure S5


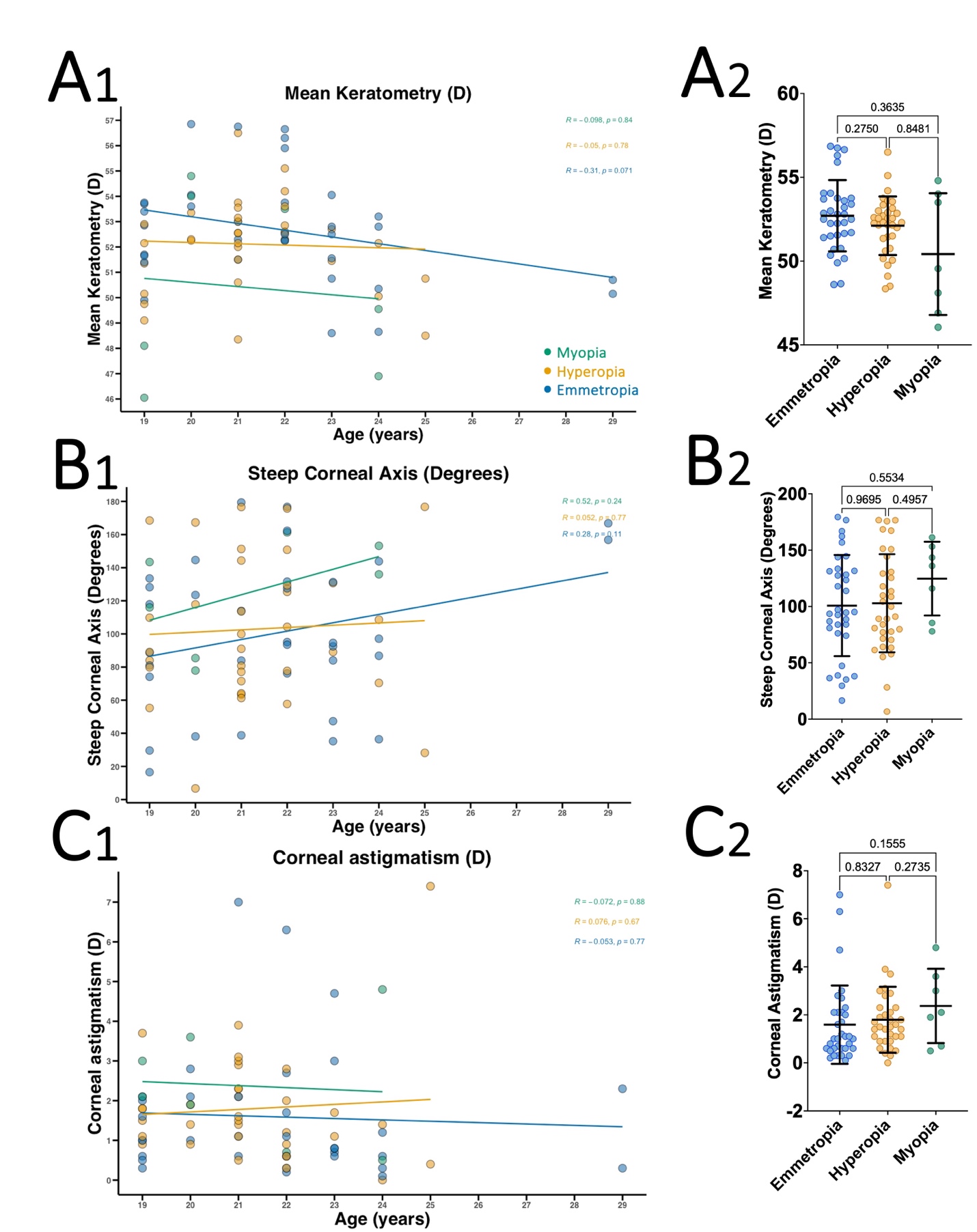


**Supplementary Figure S5. Age-related variation in corneal parameters measured by Pentacam in geriatric rhesus macaques by refractive error group. A1**, Mean keratometry (K; average keratometry reading measured in diopters) plotted as a function of age, stratified by refractive error group (myopia, hyperopia, and emmetropia). **A2**, Group-wise comparison of K values across refractive error groups. **B1**, Steep corneal axis (average location of the steepest corneal meridian in degrees) as a function of age. **B2**, Group-wise comparison of steep axis values. **C1**, Corneal astigmatism (Astig; average corneal astigmatism measured in diopters) plotted against age. **C2**, Group-wise comparison of astigmatism values. For panels **A2**, **B2**, and **C2**, group data are presented as mean ± standard deviation (SD). No significant correlations or group differences were detected for any parameter. *P*-values from statistical comparisons are indicated.

# Supplementary Table S1

**Supplementary Table S1. Distribution of Refractive Error by Environmental Housing Condition.**

| **First 6 years of life** | | | |
| --- | --- | --- | --- |
|  | **Mostly indoor** | **Mostly outdoor** | Total (n) |
| Emmetropia | 2 (8.0%) | 23 (92.0%) | 25 |
| Hyperopia | 1 (1.9%) | 52 (98.1%) | 53 |
| Myopia | 0 (0.0%) | 10 (100.0%) | 10 |
| **Overall in their life** (*P*=0.53) | | | |
|  | **Mostly indoor** | **Mostly outdoor** | Total (n) |
| Emmetropia | 12 (48.0%) | 13 (52.0%) | 25 |
| Hyperopia | 22 (41.5%) | 31 (58.5%) | 53 |
| Myopia | 6 (60.0%) | 4 (40.0%) | 10 |

Note: The animals were classified as “Mostly indoor” or “Mostly outdoor” if they spent more than 50% of the time in either environment before their refractive error was measured. *P*=0.53 (Chi-square test) indicates that there was no statistically significant association between refractive error group and lifetime environmental housing condition.
